# Tracing the mass flow from glucose and phenylalanine to pinoresinol and its glycosides in *Phomopsis* sp. XP-8 using stable isotope assisted TOF-MS

**DOI:** 10.1038/s41598-019-54836-1

**Published:** 2019-12-06

**Authors:** Yan Zhang, Junling Shi, Yongqing Ni, Yanlin Liu, Zhixia Zhao, Xixi Zhao, Zhenhong Gao

**Affiliations:** 1 Key Laboratory for Space Bioscience and Biotechnology, School of Life Sciences, Northwestern Polytechnical University, 127 Youyi West Road, Xi’an, Shaanxi Province 710072 China; 2 College of Food, Shihezi University, Road Beisi, Shihezi, Xinjiang Province 832003 China; 3 College of Enology, Northwest A & F University, Yangling, Shaanxi Province 712100 China

**Keywords:** Drug discovery, Microbiology

## Abstract

*Phomopsis* sp. XP-8, an endophytic fungus from the bark of Tu-Chung (*Eucommia ulmoides* Oliv) showed capability to biosynthesize pinoresinol (Pin) and pinoresinol diglucoside (PDG) from glucose (glu) and phenylalanine (Phe). To verify the mass flow in the biosynthesis pathway, [^13^C_6_]-labeled glu and [^13^C_6_]-labeled Phe were separately fed to the strain as sole substrates and [^13^C_6_]-labeled products were detected by ultra-high-performance liquid chromatography-quadrupole time of flight mass spectrometry. As results, [^13^C_6_]-labeled Phe was incorporated into [^13^C_6_]-cinnamylic acid (Ca) and *p*-coumaric acid (*p*-Co), and [^13^C_12_]-labeled Pin, which revealed that the Pin benzene ring came from Phe via the phenylpropane pathway. [^13^C_6_]-Labeled Ca and *p*-Co, [^13^C_12_]-labeled Pin, [^13^C_18_]-labeled pinoresinol monoglucoside (PMG), and [^13^C_18_]-labeled PDG products were found when [^13^C_6_]-labeled glu was used, demonstrating that the benzene ring and glucoside of PDG originated from glu. It was also determined that PMG was not the direct precursor of PDG in the biosynthetic pathway. The study identified the occurrence of phenylalanine- lignan biosynthesis pathway in fungi at the level of mass flow.

## Introduction

Pinoresinol diglucoside (PDG), (+)-1-pinoresinol 4, 4′-di-O-β-D-glucopyranoside, is a major antihypertensive compound found in Tu-Chung, a traditional herb medicine with excellent efficacy for lowering blood pressure^1^. PDG also possesses the potential to prevent osteoporosis^2^. Additionally, in the human intestine, PDG can be converted to enterolignans by intestinal microflora^3^, and enterolignans have potential to reduce the risk of breast cancer^4^ and other estrogen-dependent cancers^5^.

PDG is found primarily in plants as lignans^1,6^ but yields are very low. *Phomopsis* sp. XP-8 is an endophytic fungus isolated from the bark of Tu-Chung that was previously found to produce PDG *in vitro*^7^, thus, providing an alternative resource to obtain PDG. This is the first report on the capability of a microorganism to synthesize lignan. However, the PDG production by *Phomopsis* sp. XP-8 was very low, which might be enhanced by regulatory controls based on the biosynthetic pathways. Therefore, it is essential to identify the PDG biosynthetic pathway in this strain.

**Figure 1:**
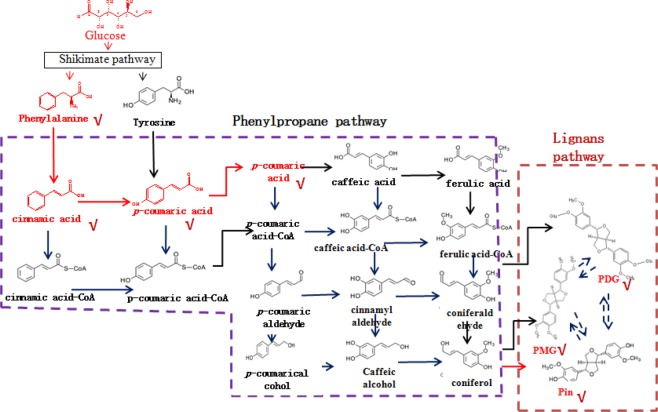
Biosynthetic pathways leading to lignans in plants. The steps which have been detected in plants were shown as solid arrow: synthesis of pinoresinol (Pin) occurs via oxidative coupling of coniferyl alcohol; coniferyl alcohol can be synthesized from glu through the phenylpropanoid pathway^10^; PMG and PDG are converted from Pin by UDP-glucose-dependent glucosyltransferase^8^. while the biosynthesis of PDG from Pin has not been detected in plants, so they are connected with dashed arrow. The substrates which have been found in the fermentation medium of *Phomopsis* sp. XP-8 in our preliminary experiment were labeled as red.

The lignan biosynthetic pathway has only been reported in plants until now^8,9^. Synthesis of Pin in plants occurs via oxidative coupling of monolignols, which are synthesized through the phenylpropanoid pathway with Phe, Ca, *p*-Co, *p*-coumaroyl-CoA, caffeate, ferulate, feruloy-CoA, coniferylaldehyde, and coniferyl alcohol as intermediates or precursors^10,11^ (Fig. 1). PMG and PDG are converted from Pin by UDP-glucose-dependent glucosyltransferase^8^. However, the biosynthesis of PDG from Pin has not been detected in plants and the Pin, PMG, and PDG biosynthetic pathways have not been elucidated in microorganisms.

We previously reported that *Phomopsis* sp. XP-8 converts mung bean starch and polysaccharides to Pin, PMG, and PDG. Phe, cinnamic acid, and *p*-coumaric acid have been detected as products of the bioconversion^12,13^. Precursor feeding and enzymatic activity measurements indicate that this strain synthesizes PDG via many steps, such as during mass flow of the phenylpropanoid pathway^14^. Genomic annotation indicates that the phenylpropane pathway exists in this strain^15^ and some other microorganisms^16^. However, the functions of the denoted genes have not been verified until now. Therefore, it is necessary to verify the entire PDG biosynthetic pathway in *Phomopsis* sp. XP-8.

Using stable or radioactive isotope-labeled compounds is an efficient and reliable strategy to verify the mass flow of unknown biosynthetic pathways by tracing the isotope-labeled compounds from substrates to products^17^. ^13^C-labeled substrates have been used to shed light on the biodegradation pathways of organic pollutants^18^. Isotope labeling combined with high-resolution mass spectrometry have also been used to track the abiotic transformation of pollutants in aqueous mixtures^19^. In recent years, liquid chromatography-mass spectrometry (LC–MS) and ultra-high-performance liquid chromatography (UPLC) systems have been developed to facilitate the analysis of many substances at the same time with high sensitivity and selectivity^20^. Stable isotope-labeled compounds have also been employed in several areas of biomedical research^21^. The combination of stable isotope-labeling techniques with MS has allowed rapid acquisition and interpretation of data and has been used in many fields, including distribution, metabolism, food, and excretion studies^22–24^. The biochemical pathway of the aromatic compounds in tea has been also been revealed using the stable isotope labeling method^25^.

In this study, we applied stable isotopes labeling and MS to trace the PDG biosynthetic pathway. Stable isotope-labeled ^13^C_6_ glu and ^13^C_6_ Phe were used as the substrates and ultra-high-performance liquid chromatography-quadrupole time of flight mass spectrometry (UPLC-Q-TOF-MS/MS) was used to identify the products.

## Materials and Methods

### Microorganism and chemicals

*Phomopsis* sp. XP-8 previously isolated from the bark of Tu-Chung and stored at the China Center for Type Culture Collection (Wuhan, China) (code: *Phomopsis* sp. CCTCC M 209291) was used in this study.

Phe (purity ≥98%, Sigma, St. Louis, MO, USA), Ca and *p*-Co (purity ≥98%; Aladdin, Shanghai, China), PDG, PMG, and Pin (purity ≥99%; National Institutes for Food and Drug Control, Beijing, China) were used as the standards (dissolved in methanol) for the structural analysis and product identification. [^13^C_6_]-Labeled Phe and glu were purchased from the Qingdao TrachinoidCo (≥99%; Qingdao, China). The purity of the [^13^C_6_]-labeled Phe and glu was 99%. Methanol (HPLC grade) was purchased from Fisher Scientific (Fairlawn, NJ, USA). The water used in the experiment was purified using a Milli-Q water purification system (18.5 M) (Millipore Corp., Bedford, MA, USA). Other reagents and chemicals were of analytical grade.

### Preparation of *Phomopsis* sp. XP-8 cells

*Phomopsis* sp. XP-8 was grown at 28 °C on potato dextrose agar plates for 5 days. Then, three pieces of mycelia (5 mm in diameter) were inoculated into 100 mL liquid potato dextrose broth in a 250-mL flask and cultivated at 28 °C on a rotary shaker (180 rpm). After 4 days, the cells were collected by centrifugation at 4 °C (1,136 × g for10 min) using a centrifuge (HC-3018R, Anhui USTC Zonkia Scientific Instruments Co., Ltd., Anhui, China). The cells were washed twice with sterile water and used for bioconversion according to the experimental design.

### Bioconversion systems

The bioconversion with unlabeled glu as the sole substrate was carried out in a 250-mL flask containing 100 mL of ultrapure water (pH 7), 5 g/L glu, and the prepared *Phomopsis* sp. XP-8 cell set a ratio of 10 g cells (wet weight) per 100 mL medium. To track the mass flow from glu to PDG, glu was changed to 5 g/L [^13^C_6_]-labeled glu in the above medium and the same conditions were used for bioconversion.

Bioconversion with Phe as the sole substrate was carried out in medium without glu, 7 mM [^13^C_6_]-labeled phenylalanine, and the prepared *Phomopsis* sp. XP-8 cells at a ratio of 10 g wet cells per 100 mL medium.

All bioconversions were carried out for 48 h at 28 °C and 180 rpm. At the end of bioconversion, the broth was collected and filtered through an intermediate speed qualitative filter paper before the products were detected.

### Identification of the accumulated products during bioconversion

The products were extracted from the vacuum-evaporated (0.09 MPa, 50 °C) bioconversion broth with methanol and adjusted to 4 mL for the UPLC measurements after filtration through a membrane (0.45 µm, 13 mm diameter; Millipore, Billerica, MA, USA). The UPLC analysis was performed on a Waters Acquity UPLC system (Waters Corp., Milford, MA, USA), equipped with a binary pump, a thermostatically controlled column compartment, and a UV detector. Gradient elution was performed on an Acquity UPLCTM BEH C18 column (50 mm × 2.1 mm I.D., 1.7 m; Waters) and the column temperature was maintained at 30 °C, while sample temperature was 10 °C^13^.

The MS analysis of the products was carried out on a Q-TOF Premier^TM^ with an ESI source (Waters Corp.) at the optimized parameters of: capillary voltage, 2.8 kV; sampling cone voltage, 20 V; extractor voltage, 4 V; source temperature, 100 °C; desolvation temperature, 250 °C, and flow rate of the desolvation gas (N_2_), 400 L/h. The collision cell parameters for the Q-TOF-MS/MS analysis were: collision gas (Argon) flow rate, 0.45 L/h; collision energy, 15–35 eV. The mass spectra were recorded using full scan mode over a mass range of *m/z* 100–800 in negative ion mode. The MS acquisition rate was set to 1.0 s, with a 0.02 s interscan delay. The Q-TOF-MS/MS experiments were carried out by setting the quadrupole to allow ions of interest to pass prior to fragmentation in the collision cell.

Accurate mass measurements were obtained by means of a lock mass that introduces a low flow rate (3 L/min) of a chrysophanol (253.0499) calibrating solution in the ESI-Q-TOF-MS and ESI-Q-TOF-MS/MS. All operations and acquisition and data analyses were controlled by Masslynx V4.1 software (Waters Corp.).

### Data processing

Peak detection, alignment, and identification of the detected compounds were performed using Masslynx V4.1 software (Waters Corp.). The MS/MS fragmentation patterns were used for informative non-targeted metabolic profiling of the LC-MS data, and the acquired LC-MS/MS spectrum was identified after comparison with spectra proposed by the Mass bank database (www.massbank.jp), the KEGG database, and related reports.

## Results

### Detection of products converted from unlabeled glu

**Figure 2:**
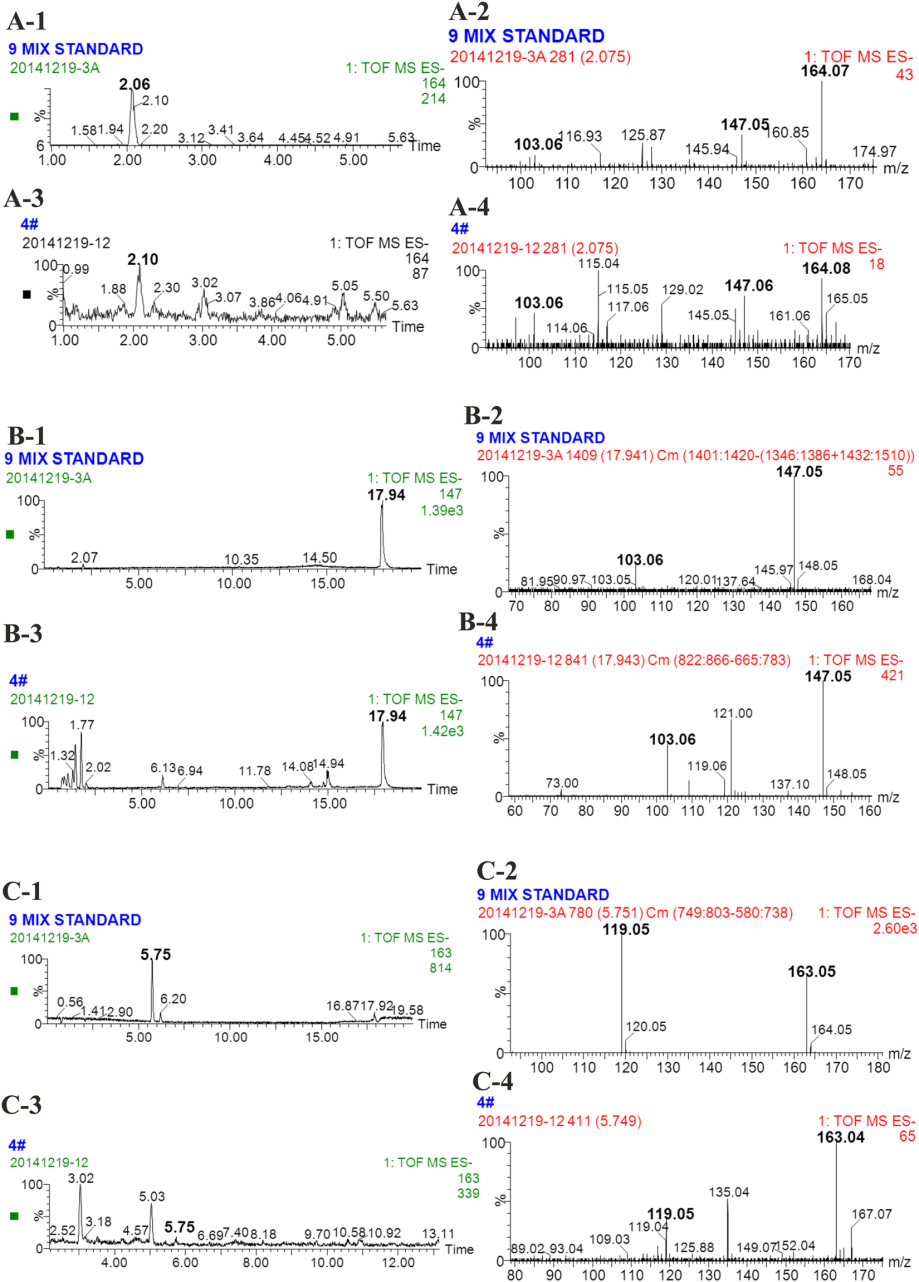
Total ion current chromatogram and mass spectrum of phenylalanine (**A**), cinnamic acid (**B**), *p*-coumaric acid (**C**). (A-1, B-1, and C-1 show the total ion current chromatogram of standard phenylalanine, cinnamic acid and *p*-coumaric acid respectively; A-2, B-2, and C-2 show mass spectrum of standard phenylalanine, cinnamic acid and *p*-coumaric acid respectively; A-3, B-3, and C-3 show the total ion current chromatogram of phenylalanine, cinnamic acid and *p*-coumaric acid in the samples, respectively; A-4, B-4, and C-4 show mass spectrum of phenylalanine, cinnamic acid and *p*-coumaric acid in the samples, respectively. Ion reaction were set to *m/z* = 164, *m/z* = 147 and *m/z* = 163 respectively.)

**Figure 3:**
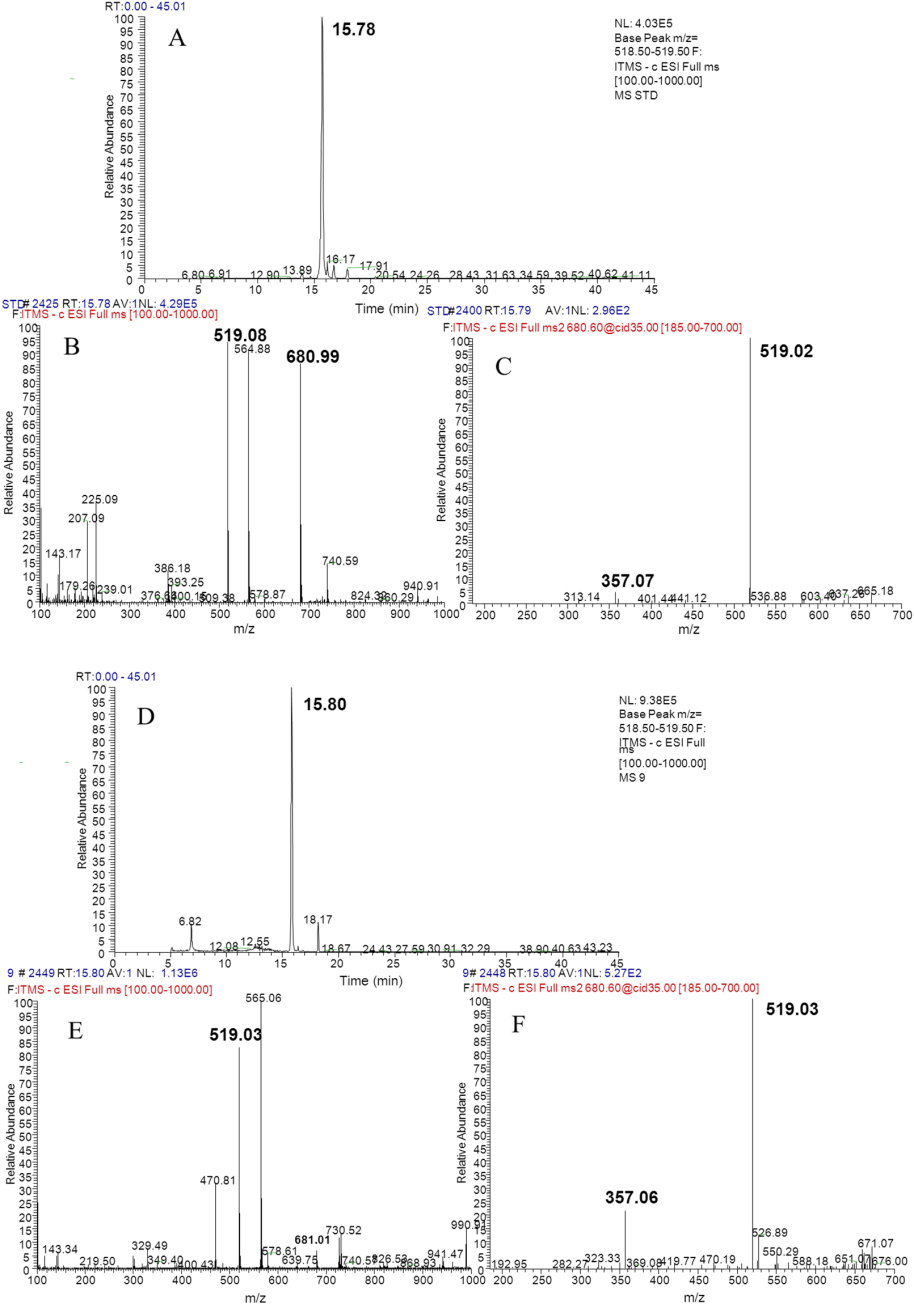
Total ion current chromatogram and mass spectrum of PDG (**A**–**C** are the total ion current chromatogram, precursor ions, and daughter ions of standard PDG, respectively; **D**–**F** are the total ion current chromatogram, precursor ions, and daughter ions of the samples, respectively).

**Figure 4:**
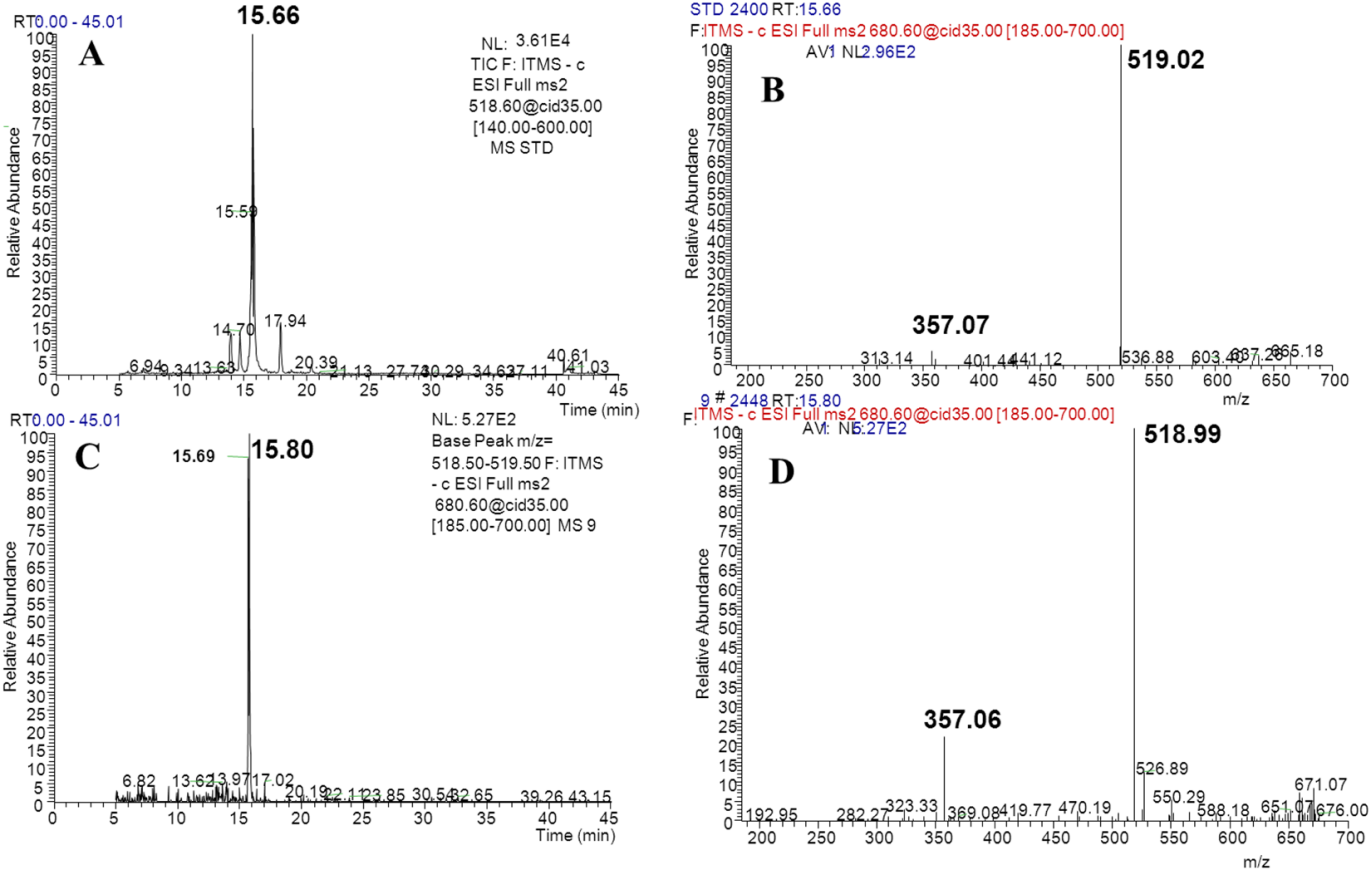
Total ion current chromatogram and mass spectrum of PMG (**A**,**B** show the total ion current chromatogram and mass spectrum of standardpinoresinol-4-O-β-D-glucopyranoside, respectively; **D**–**F** show the total ion current chromatogram and the mass spectrum ofpinoresinol-4-O-β-D-glucopyranosideinsamples, respectively. Ion reaction was set to *m/z* = 518.5–519.5).

**Figure 5:**
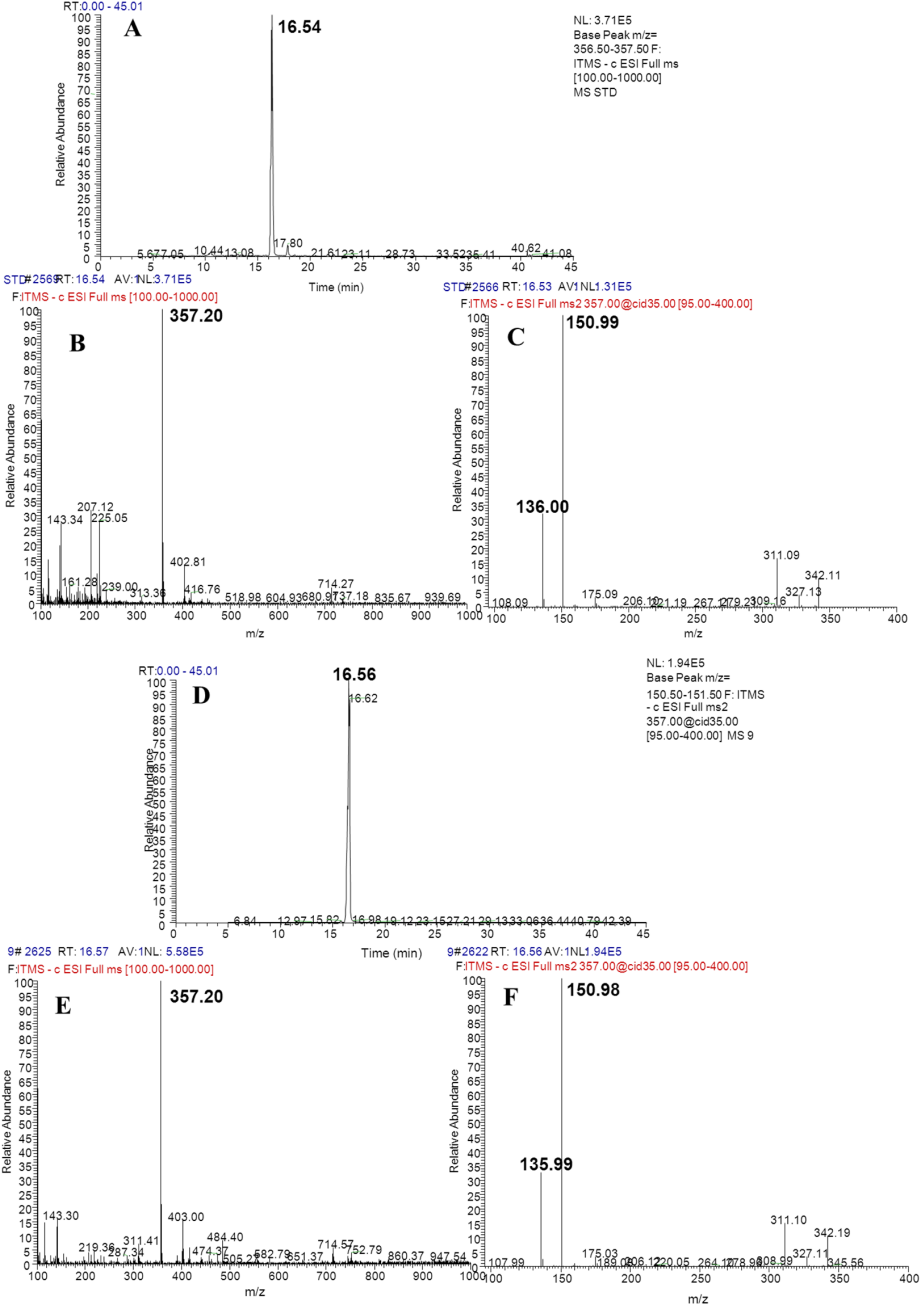
Total ion current chromatogram and mass spectrum of Pin (**A**–**C** show the total ion current chromatogram, precursor ions, and daughter ions of the Pin standard, respectively; **D**–**F** show the total ion current chromatogram, precursor ions, and daughter ions of Pin in the samples, respectively. Ion reaction was set to *m/z* = 356.5–357.5).

Production of PDG, PMG, Pin, Phe, *p*-Co, and Ca were detected in bioconversion systems using glu as the sole substrate. Data in Figs. 2–5 show the mass spectra of these compounds accumulated in the bioconversion systems and the corresponding standards.

Production of Phe was detected as *m/z* = 164.08, and *m/z* = 147.06 (Fig. 2A-4), which was consistent with the data obtained from the corresponding standards (Fig. 2A-2). Similarly, production of PDG, PMG, Pin, *p*-Co, and Ca was also detected in the bioconversion system, indicating that glu was converted to these products by *Phomopsis* sp. XP-8, as only glu was provided in the bioconversion system.

### Identification of products converted from [^13^C_6_]-labeled Phe

**Figure 6:**
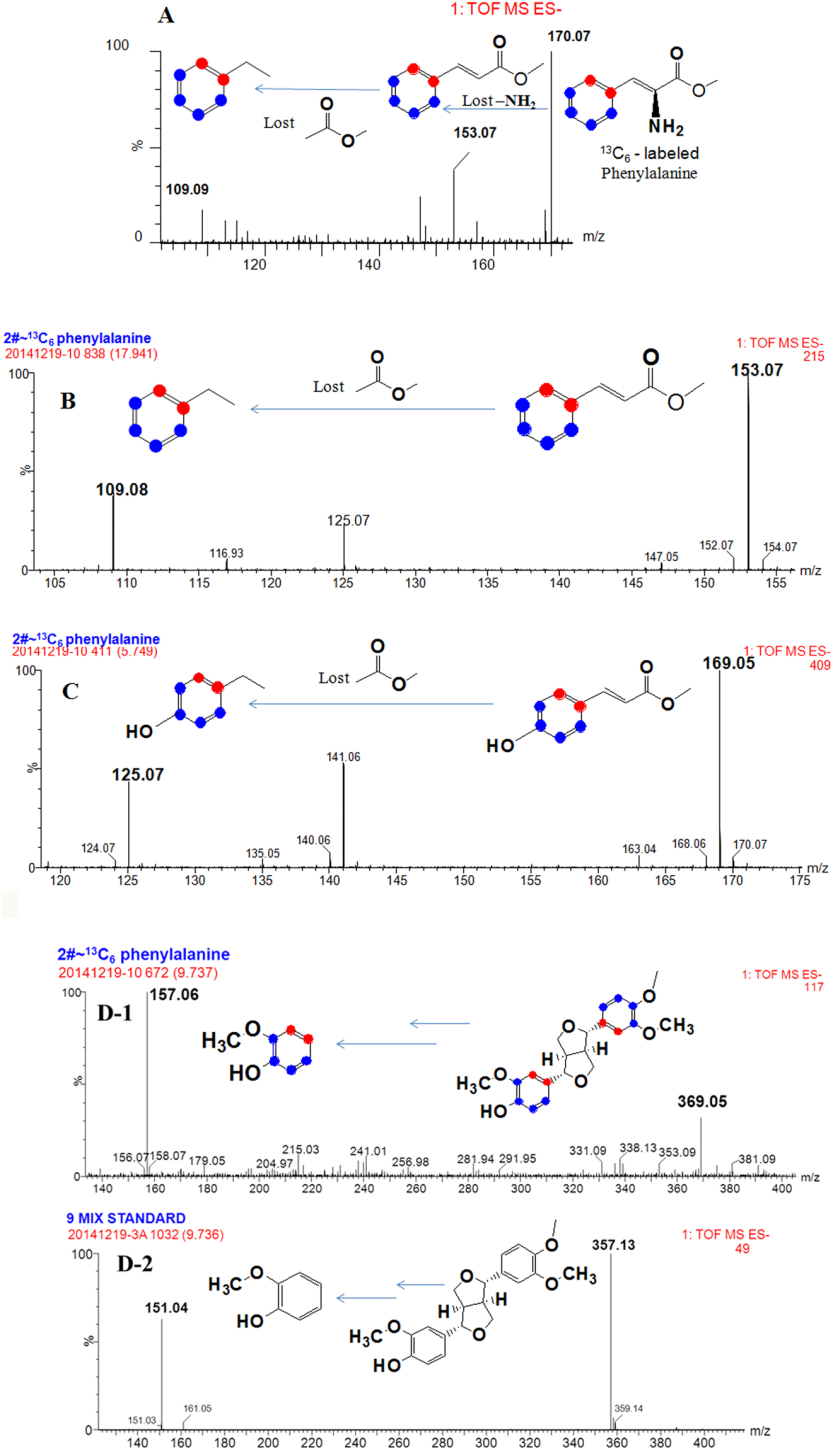
Mass spectrum of phenylalanine (**A**), cinnamic acid (**B**), *p*-coumaric acid (**C**), and Pin (**D**) in the resting cell system using [^13^C_6_]-labeled phenylalanine as the substrate.

The phenylpropanoid pathway in plants starts with Phe and ends with *p-*Co. The same mass flow was previously detected during PDG biofrom glu by *Phomopsis* sp. XP-8^13^. To verify this finding and the role of the Phe pathway in the biosynthesis of PDG, PMG, and Pin, [^13^C_6_]-labeled Phe was used as the sole substrate in the bioconversion system without glu (mainly used as the glucoside donor). As results, ^13^C labeled Pin, Phe, *p*-Co, and Ca were successfully detected (Fig. 6). The products were successfully detected at the same RT of their corresponding unlabeled standard substrates. All ^13^C-labeled product data and their corresponding standard substrates are summarized in Table S1 (Supporting information).

As shown in Fig. 6B, ^13^C-labeled Ca was detected as *m/z* = 153.07, indicating that six ^13^C from [^13^C_6_]-labeled Phe were incorporated into Ca. A daughter ion of ^13^C-labeled Ca was obtained at *m/z* = 109.08, indicating six ^13^C referring to the standard Ca (*m/z* = 103.06). The structure of ^13^C-labeled Ca without -COO^−^ was observed at *m/z* = 109.08. Therefore, it was deduced that the six ^13^C were incorporated into the benzene ring of Ca not into –COO^−^.

*P*-Co produced in the conversion system was detected as *m/z* = 169.05 and revealed six ^13^C by consulting the *p*-Co standard (Fig. 6C). A daughter ion of ^13^C-labeled *p*-Co was obtained at *m/z* = 125.07, indicating 6 Da mass shift than *p*-Co standard (*m/z* = 119.06). The structure of ^13^C-labeled *p*-Co without –COO^−^ (44 Da lost) was observed at *m/z* = 125.07. Therefore, it was deduced that the six ^13^C might be distributed in the benzene ring.

^13^C-labeled Pin was detected (Fig. 6D-1) and compared with the mass spectra of the Pin standard (C_20_H_22_O_6_, RT = 9.736 min, detected as *m/z* = 357.13 and *m/z* = 151.04 respectively) (Table S2, Supporting information). ^13^C-labeled Pin was detected as *m/z* = 369.05, indicating 12 Da mass shift than Pin standard (*m/z* = 357.13). A daughter ion of ^13^C-labeled Pin was observed at *m/z* = 157.06, which showed a mass increase of 6 Da than Pin standard (*m/z* = 151.04). The structure of ^13^C-labeled Pin with loss of a benzene ring was identified as the major daughter ion of *m/z* = 157.06 (Fig. 6D-1). This result confirmed that the six^13^C were distributed in a benzene ring, whereas the other six^13^C might be in a symmetrical benzene ring. Therefore, we deduced that the Pin with 12 ^13^C was bio-converted from the [^13^C_6_]-labeled Phe, Ca, or/and *p*-Co. This finding also confirmed that the benzene ring in Pin came from Phe, which is consistent with that of the lignan biosynthetic pathway in plants.

### Identification of products converted from [^13^C_6_]-labeled glucose

**Figure 7:**
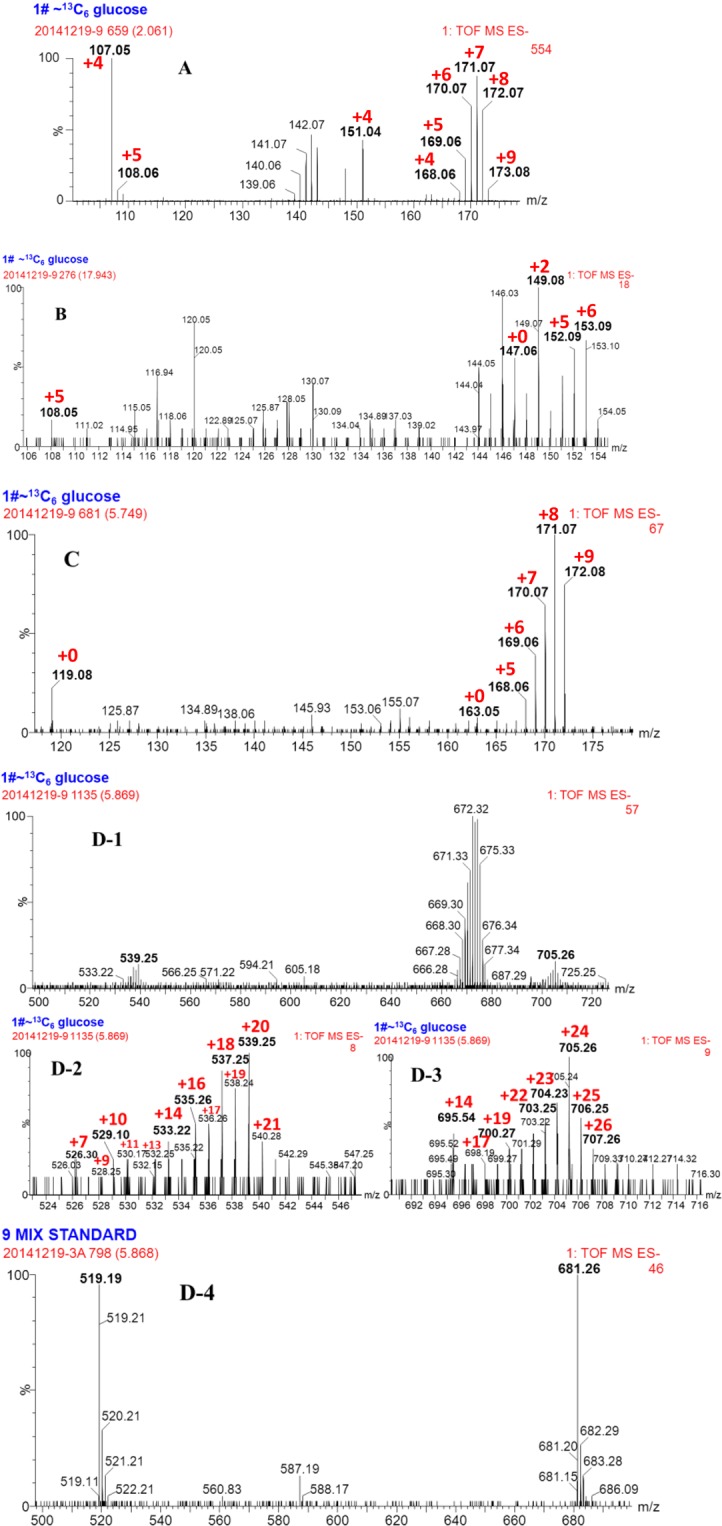
Mass spectrum of phenylalanine (**A**), cinnamic acid (**B**), *p*-coumaric acid (**C**) and PDG (**D**) in the resting cell system using [^13^C_6_]-labeled glucose as the substrate. The increase of *m/z* owing to incorporation of ^13^C is shown in red.

**Figure 8:**
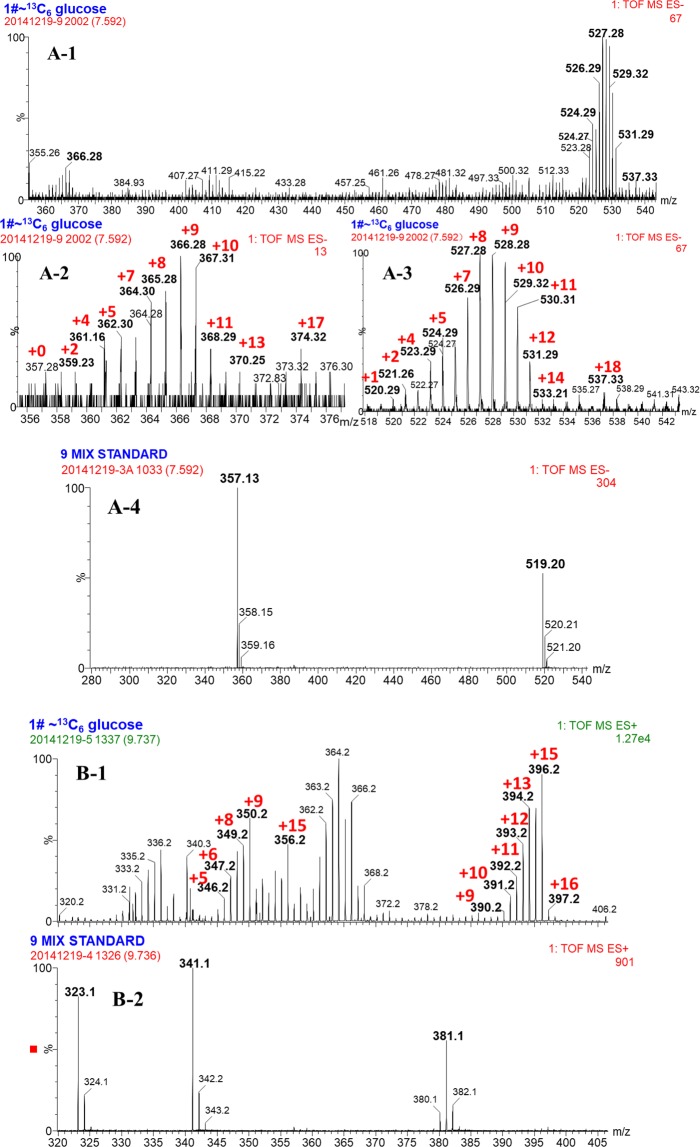
Mass spectrum of PMG (**A**) and Pin (**B**) in the resting cell system using [^13^C_6_]-labeled glucose as the substrate. The increase of *m/z* owing to incorporation of ^13^C is shown in red.

To explore where Phe originated from the Pin biosynthetic pathway, [^13^C_6_]-labeled glu was supplied as the sole substrate in the bioconversion system with *Phomopsis* sp. XP-8 cells. As results, ^13^C labeled PDG, PMG, Pin, Phe, *p*-Co, and Ca were detected (Figs. 7 and 8).

The isotopic patterns observed in the MS and MS/MS spectra suggest the ^13^C from ^13^C_6_-labeled glu were incorporated into the products of Phe (Fig. 7A), Ca (Fig. 7B) *p*-Co (Fig. 7C), PDG (Fig. 7D), PMG (Fig. 8A), Pin (Fig. 8B), respectively. The observed mass shifts, indicating the number of incorporated ^13^C, were shown in the spectra. The detailed information on the products and possible positions of ^13^C in the products are summarized in Table S2 (Supporting information).

Interestingly, the analysis revealed that ^13^C_6_-labeled glu were incorporated into the core structure of PDG and PMG, and their glycosides. Additionally, the maximum of 16 ^13^C was detected in the formed Pin (C_20_H_22_O_6_), indicating the [^13^C_6_]-labeled glu partly contributed to the formation of Pin. The possible positions of ^13^C in the structures are summarized in Table S2 (Supporting information).

**Figure 9:**
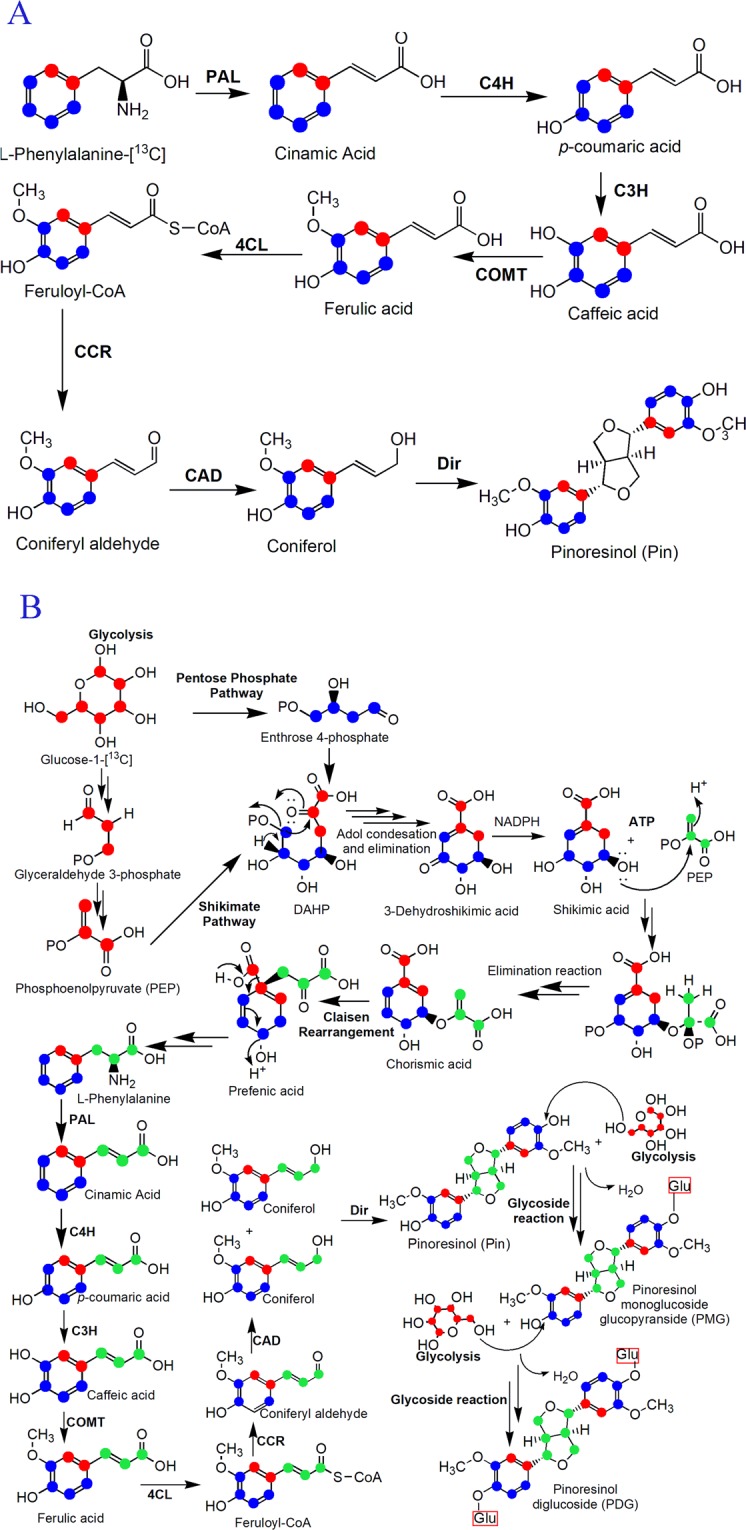
Pin and PDG bioconversion pathway in *Phomopsis* sp. XP-8. (**A**: Pin biosynthesis scheme from ^13^C stable isotope labeled phenylalanine; **B**: PDG biosynthesis scheme from ^13^C stable isotope labeled glucose). The abbreviations indicate phenylalanine ammonia-lyase (PAL), trans-cinnamate 4-hydroxylase (C4H), 4-coumarate-CoA ligase (4CL), p-coumarate 3-hydroxylase (C3H), caffeic acid 3-O-methyltransferase (COMT), cinnamoyl-CoA reductase (CCR), carbamyl phosphate synthetase (CAD), dirigent protein (Dir). ^13^C stable isotopes were signed by coloured dots. Among the dots, red dots mean that the ^13^C stable isotopes were converted from ^13^C isotopes labeled glucose through Phosphoenolpyruvate (PEP); blue dots mean that the ^13^C isotopes were converted from ^13^C isotopes labeled glucose through Enthrose 4-phosphate; green dots means that the ^13^C stable isotopes were converted from another ^13^C isotopes labeled glucose through the intermediate substances of PEP.

Taken together, the mass flow from [^13^C_6_]- Phe to [^13^C_6_]-Ca, [^13^C_6_]-*p*-Co, and [^13^C_12_]-Pin was verified by the experiments using [^13^C_6_]-labeled Phe as the sole substrate (Fig. 9A). The mass flow from [^13^C_6_]- glu to [^13^C]-Phe, [^13^C]-Ca, [^13^C]-*p*-Co, [^13^C]-Pin, [^13^C]-PMG, and[^13^C]-PDG was verified by the data obtained using [^13^C_6_]- glu as the sole substrate (Fig. 9B).

### Possible pathways for biosynthesis of PDG and PMG

**Figure 10:**
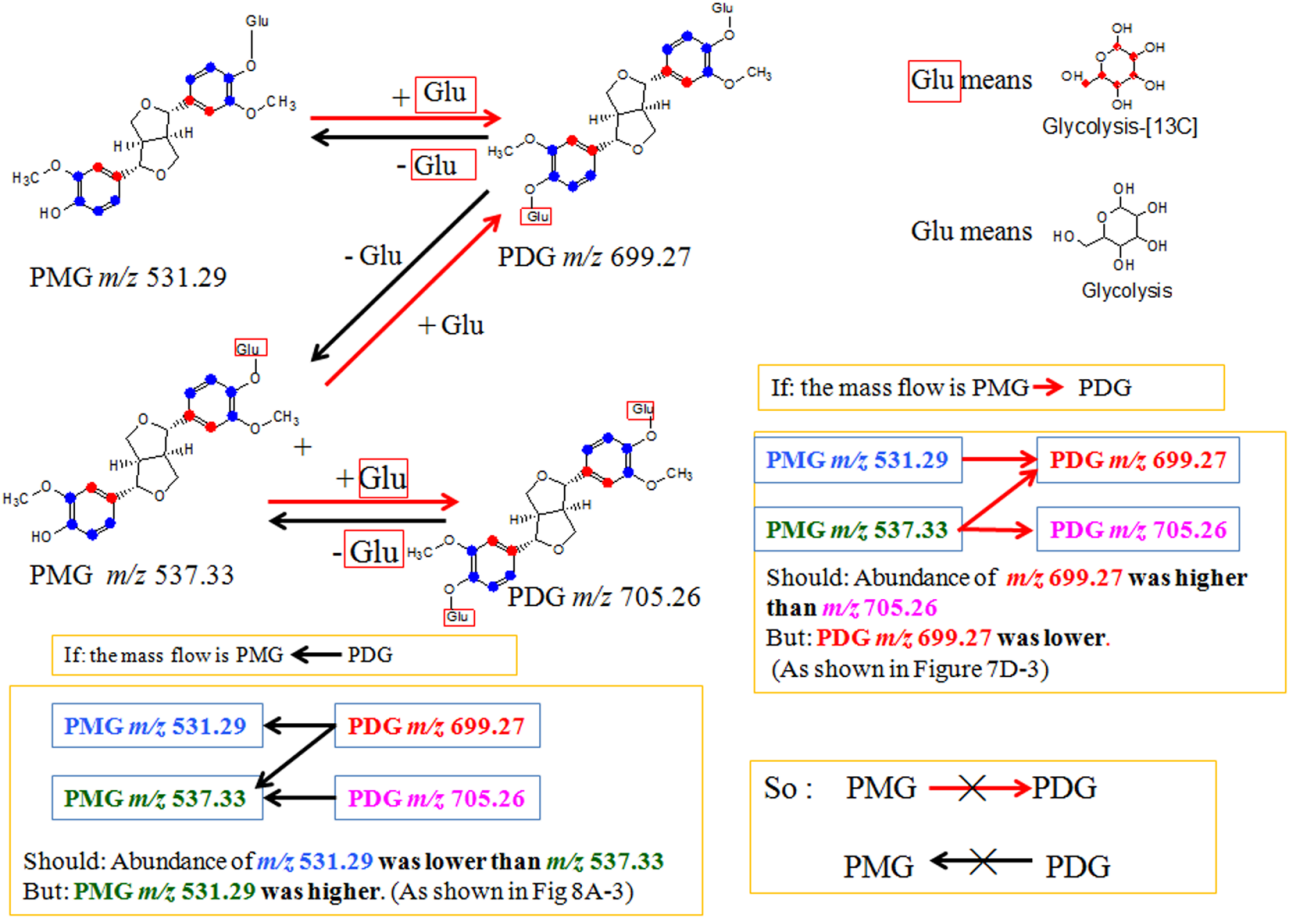
Bioconversion between PDG and PMG in *Phomopsis* sp. XP-8. Glu with red box means ^13^C_6_ labeled glycoside, Glu without red box means unlabeled glycoside. “×”means the pathway does not exist in *Phomopsis* sp. XP-8.

The evidences for the possible biosynthetic pathways of PDG, PMG, and Pin are summarized in Figs. 9 and 10. The pathway from Phe to Pin, glu to Phe, Pin, PMG and PDG was verified (Fig. 9A,B, Supporting information Table S1 and Table S1). In addition, the bioconversion between PDG and PMG in *Phomopsis* sp. XP-8 was reported for the first time, and the analysis was showed in Fig. 10.

As shown in Fig. 10, two structures of PMG were detected: one was [^13^C_12_]-PMG with two benzene rings converted from ^13^C-labeled glu and an unlabeled glycoside (PMG *m/z* 531.29), and the other was [^13^C_18_]-PMG with both benzene ring structures converted and a glucoside from ^13^C-labeled glu (PMG *m/z* 537.33). Similarly, two PDG structures were detected: one was [^13^C_18_]-PDG with a two benzene ring structure and one glycoside converted from ^13^C-labeled glu (PDG *m/z* 699.27); the other one was [^13^C_24_]- PDG with two benzene rings and two glycosides from ^13^C-labeled glu (PDG *m/z* 705.26).

If PMG was the direct precursor of PDG, PMG *m/z* 531.29 would be converted to PDG *m/z* 699.27 by bonding one [^13^C_6_]-labeled glycoside through glycosylation; PMG *m/z* 537.33 could also be converted to PDG *m/z* 699.27 by bonding one unlabeled glycoside through glycosylation and to PDG *m/z* 705.26 by bonding one [^13^C_6_]-labeled glycoside. If this is true, PDG *m/z* 699.27 would have two glucoside sources, whereas PDG *m/z* 705.26 would have only one glucoside source. Therefore, the concentration of PDG *m/z* 705.26 should be lower than PDG *m/z* 699.27. However, the data show that the relative abundance of PDG *m/z* 705.26 was much higher than that of PDG *m/z* 699.27 (Fig. 7D-3). Therefore, PMG was not the precursor of PDG.

In contrast, if PDG was the direct precursor of PMG, PDG *m/z* 699.27 would be converted to PMG *m/z* 531.29 by hydrolyzation of one [^13^C_6_]-labeled glycoside and to PMG *m/z* 537.33 by hydrolyzation of one unlabeled glycoside; PDG *m/z* 705.26 would be converted to PMG *m/z* 537.33 by hydrolyzation of one [^13^C_6_]-labeled glycoside. If this is true, PMG *m/z* 537.33 would have two glycoside sources, whereas PMG *m/z* 531.29 would have only one source. The concentration of PMG *m/z* 531.29 should be lower than PMG *m/z* 537.33. However, the data show that relative abundance of *m/z* = 531.29 was higher than that of *m/z* = 537.33 (Fig. 8A-3). Therefore, PDG was not the precursor of PMG.

## Discussion

The ^13^C stable isotope labeling method was successfully used in this study to verify the phenylpropanoid-pinoresinol and biosynthetic pathway of its glycosides in *Phomopsis* sp. XP-8 during mass flow. This lignan biosynthetic pathway was only reported in plants until now^8,9^, so it was very significant to verify the occurrence of this pathway in microorganisms. Stable Isotope-assisted metabolomics is an efficient way to trace and identify bio-transformed products and the metabolic pathways involved in their formation, such as understanding the fate of organic pollutants in environmental samples^17^. It was the first time to use this method to verify the Phenylpropanoid-pinoresinol in a microorganism. In our previous studies, many methods such as precursor feeding^13^, detection of enzyme activity^14^, and genomic annotation^15^ have been used to analyze the Phenylpropanoid-pinoresinol biosynthetic pathway in *Phomopsis* sp. XP-8. Through these studies, the precursors, enzymes activity and genes of PDG biosynthetic pathway have been found. The ^13^C stable isotope labeling method gave further verification on the occurrence of lignan biosynthetic pathway in microorganisms by now. In addition to this, it is the first time that differences between the PDG and PMG biosynthetic pathways have been verified.

The results obtained in this study verify the existence of the phenylpropanoid-lignan metabolic pathway in *Phomopsis* sp. XP-8. Genomic annotation is an efficient way to discover the pathways that are normally difficult to reveal by metabolic and enzymatic evidence due to low intermediate accumulation, low end-product, and silent gene expression under normal conditions. This method has been successfully used to identify the existence of a phenylpropanoid metabolic pathway in *Aspergillus oryzae*^26^, and the molecular genetics of naringenin biosynthesis, a typical plant secondary metabolite in *Streptomyces clavuligerus*^27^, and the occurrence of the phenylpropanoid-lignan pathway in *Phomopsis* sp. XP-8^15^. This study reports the existence of the phenylpropanoid-lignan pathway in *Phomopsis* sp. XP-8 during mass flow and identified the metabolites.

Additional studies should illustrate the origin of the genes in the phenylpropanoid-lignan pathway of *Phomopsis* sp. XP-8. Horizontal gene transfer (HGT) has long been recognized as an important force in the evolution of organisms^28^. HGT occurs among different bacteria and plays important roles in the adaptation of microorganisms to different hosts or environmental conditions^29^. More and more evidence for gene transfer between distantly related eukaryotic groups has been presented^28^.Therefore, we cannot exclude the possibility that XP-8 may have acquired the genes related to the lignan biosynthetic pathway from its host plant by HGT during long-term symbiosis and evolution. However, further evidence is still needed to verify this proposed process.

The results obtained in this study provide useful information on the biosynthesis of lignans and their glycosides via microbial fermentation. Biosynthesis of lignans is of great interest to organic chemists as it provides a model for biomimetic chemistry and has extensive applications^30^. Improvement has been made in the techniques to biosynthesize lignan products by regulating the lignan biosynthetic pathway in trees through genetic modifications^31^. However, the lignan biosynthetic pathway has rarely been reported. More importantly, the bioconversion sequence from Pin to PDG and the direct precursor of PDG have remained unclear until now. In previous studies on *Phomopsis* sp. XP-8, the highest production of PDG and PMG did not occur simultaneously^12^ and PMG was not the precursor of PDG because PDG production decreased and/or disappeared when PMG yield increased^13^. The present study demonstrated that PMG was not the precursor of PDG, and PDG was not the precursor of PMG, indicating that Pin might be converted to PMG and PDG via two different pathways in *Phomopsis* sp. XP-8, which has not been revealed in plants.

Furthermore, this study revealed that the bioconversion of Pin, PMG, and PDG from glu occurred simultaneously as that from Phe. We found that the benzene ring structure of Phe did not open throughout the entire Pin bioconversion process in *Phomopsis* sp. XP-8 when Phe was used as the sole substrate, indicating that the Pin benzene ring originated from Phe. Glu was converted to Phe and was the sole glycoside donor for PDG biosynthesis. Therefore, glu not only participated in the formation of glycosides in PDG, but also provided the PDG benzene ring structure. This is different from that found in plants, indicating there might be some other different pathways to produce these products in *Phomopsis* sp. XP-8.

Not all intermediates in the KEGG-identified plant-lignan biosynthetic pathway related to Pin, PMG, and PDG formation were found in *Phomopsis* sp. XP-8, such as caffeic acid, ferulic acid, and coniferyl alcohol (Fig. 1). This may be because the pathways after *p*-Co are different in XP-8 from those in plants, or the accumulation of these intermediates was too low to be detected. Further studies are needed to verify this hypothesis.

In conclusion, the capability of *Phomopsis* sp. XP-8 to biosynthesize Pin, PMG and PDG from [^13^C_6_]-Phe and [^13^C_6_]-glu was verified. The study illustrated the phenylpropanoid-pinoresinol biosynthetic pathway in microorganism by using stable isotope assisted UPLC-Q-TOF-MS/MS, thus, demonstrating a completely new way to produce Pin, PMG and PDG by bioconversion process. In the further studies, *Phomopsis* sp. XP-8 could be used in producing these lignans and their derivatives by microbial fermentation or enzymatic reaction. In addition, the microbial fermentation production of Pin, PMG and PDG could be enhanced by regulatory controls based on the biosynthetic pathways proved in this study.

## Supplementary information

Supporting information
