# Supplementary material for: Tracing the mass flow from glucose and phenylalanine to pinoresinol and its glycosides in Phomopsis sp. XP-8 using stable isotope assisted TOF-MS
Source: Sci Rep. 2019 Dec 6;9:18495. doi: 10.1038/s41598-019-54836-1 (PMC6897942; doi:10.1038/s41598-019-54836-1)
Supplement: Supplementary file 1 — Supporting information [file 41598_2019_54836_MOESM1_ESM.docx]

Tracing the mass flow from glucose and phenylalanine to pinoresinol and its glycosides in *Phomopsis* sp. XP-8 using stable isotope assisted TOF-MS

Yan Zhang^b^, Junling Shi^a*^, Yongqing Ni^b^, Yanlin Liu^c^, Zhixia Zhao^b^, Xixi Zhao^a^, Zhenhong Gao^a^

^a^Key Laboratory for Space Bioscience and Biotechnology, School of Life Sciences, Northwestern Polytechnical University, 127 Youyi West Road, Xi’an, Shaanxi Province 710072, China.

^b^College of Food，Shihezi University, Road Beisi, Shihezi Xinjiang Province 832003,  China.

^c^College of Enology, Northwest A & F University, Yangling Shaanxi Province 712100, China.

^*^Corresponding author. Tel. +86-29-88460541; Fax. +86-29-88460541; E-mail: sjlshi2004@nwpu.edu.cn

**Table S1** Predicted products with [^13^C_6_]-labeled phenylalanine as the substrate.

| [Retention time](http://dict.youdao.com/w/retention%20time/#keyfrom=E2Ctranslation)（min） | Product information | | | [Predicted](http://dict.youdao.com/w/predicted/#keyfrom=E2Ctranslation) isotopic product | | Structure (The possible position of the labeled ^13^C were showed red) |
| --- | --- | --- | --- | --- | --- | --- |
|  | Product  [molecularformula](http://www.so.com/link?url=http%3A%2F%2Fdict.youdao.com%2Fsearch%3Fq%3D%255B%25E5%258C%2596%25E5%25AD%25A6%255D%2520molecular%2520formula%26keyfrom%3Dhao360&q=%E5%88%86%E5%AD%90%E5%BC%8F+%E8%8B%B1%E6%96%87&ts=1521261267&t=77bc39105da3077ffee03760182fd27) | Normal ^12^C  (m/z) | Structure | Heavy ^13^C  (m/z) | △M |  |
| 2.06 | Phe  C_9_H_11_NO_2_ | Major ion  164.08 | 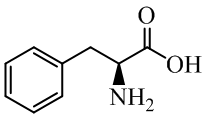 | 179.06 | 6 |  |
|  |  | Daughter ion1  147.06 | 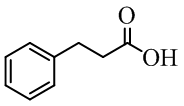 | 153.06 | 6 |  |
|  |  | Daughter ion2  103.06 |  | 109.08 | 6 |  |
| 17.94 | Ca  C_9_H_8_O_2_ | Major ion  147.05 |  | 153.07 | 6 |  |
|  |  | Daughter ion 103.06 |  | 109.08 | 6 |  |
| 5.75 | *p*-Co  C_9_H_8_O_3_ | Major ion  163.05 |  | 169.05 | 6 |  |
|  |  | Daughter ion 119.05 |  | 125.07 | 6 |  |
| 9.736 | Pin  C_20_H_22_O_6_ | Major ion  357.13 |  | 369.05 | 12 |  |
|  |  | Daughter ion 151.04 |  | 157.06 | 6 |  |

The abbreviations in the table mean phenylalanine (Phe), cinnamic acid (Ca), *p*-Coumaric acid (*p*-Co ), pinoresinol (Pin).

^13^C stable isotopes were signed by coloured dots. Among the coloured dots, red dots mean that the ^13^C stable isotopes were tracing to the Phosphoenolpyruvate (PEP) converted from ^13^C isotopes labeled glucose, blue dots mean that the ^13^C isotopes were tracing to Enthrose 4-phosphate converted from ^13^C isotop labeled glucose, green dots means that the ^13^C stable isotopes were converted from another ^13^C isotopes labeled glucose through the intermediate substances of PEP as shown in Figure 10 A,B.

**Table S2** Predicted products with [^13^C_6_]-labeled glucose as the substrate.

| [Retention time](http://dict.youdao.com/w/retention%20time/#keyfrom=E2Ctranslation)  （min） | Product information | | | [Predicted](http://dict.youdao.com/w/predicted/#keyfrom=E2Ctranslation) isotopic product | | Structure (The possible position of the labeled ^13^C were showed red) | | | | | |
| --- | --- | --- | --- | --- | --- | --- | --- | --- | --- | --- | --- |
|  | Product  [molecularformula](http://www.so.com/link?url=http%3A%2F%2Fdict.youdao.com%2Fsearch%3Fq%3D%255B%25E5%258C%2596%25E5%25AD%25A6%255D%2520molecular%2520formula%26keyfrom%3Dhao360&q=%E5%88%86%E5%AD%90%E5%BC%8F+%E8%8B%B1%E6%96%87&ts=1521261267&t=77bc39105da3077ffee03760182fd27) | Normal ^12^C  (m/z) | Structure | Heavy ^13^C  (m/z) | △M |  |  |  |  |  |  |
| 2.06 | Phe  C_9_H_11_NO_2_ | Major ion  164.08 | 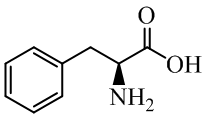 | 168.06 | 4 |  | | | | | |
|  |  |  |  | 169.06 | 5 |  | | | | | |
|  |  |  |  | 170.07 | 6 |  | | | | | |
|  |  |  |  | 171.07 | 7 |  | | | | | |
|  |  |  |  | 172.07 | 8 |  | | | | | |
|  |  |  |  | 173.08 | 9 |  | | | | | |
|  |  | Daughter ion 1  103.06 | 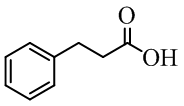 | 151.04 | 4 |  | | | | | |
|  |  | Daughter ion 2  147.06 |  | 107.05 | 4 |  | | | | | |
|  |  |  |  | 108.06 | 5 | May be interfered by the other products. | | | | | |
| 17.94 | Ca  C_9_H_8_O_2_ | Major ion  147.05 |  | 147.06 | 0 | - | | | | | |
|  |  |  |  | 149.08 | 2 |  |  | | |  | |
|  |  |  |  | 152.09 | 4 |  | | |  | | |
|  |  |  |  | 153.09 | 5 |  | | |  | |  |
|  |  |  |  | 154.05 | 6 |  | | |  | |  |
|  |  | Daughter ion 103.06 |  | 108.05 | 5 | May be interfered by the other products. | | | | | |
| 5.75 | *p*-Co  C_9_H_8_O_3_ | Major ion  163.05 |  | 163.05 | 0 | - | | | | | |
|  |  |  |  | 168.06 | 5 |  | | |  | |  |
|  |  |  |  | 169.06 | 6 |  | | |  | |  |
|  |  |  |  | 170.07 | 7 |  | | |  | |  |
|  |  |  |  | 171.07 | 8 | May be interfered by the other products. | | | | | |
|  |  |  |  | 172.08 | 9 |  | | | | | |
|  |  | Daughter ion 119.05 |  | 119.08 | 0 | - | | | | | |
| 5.868 | PDG  C_32_H_42_O_16_ | Major ion 681.26 |  | 695.54 | 14 |  | | | | | |
|  |  |  |  | 698.19 | 17 |  | | | | | |
|  |  |  |  | 699.27 | 18 |  | | | | | |
|  |  |  |  | 700.27 | 19 |  | | | | | |
|  |  |  |  | 701.29 | 20 |  | | | | | |
|  |  |  |  | 703.25 | 22 |  | | | | | |
|  |  |  |  | 704.23 | 23 |  | | | | | |
|  |  |  |  | 705.26 | 24 |  | |  | | |  |
|  |  |  |  | 706.25 | 25 |  | |  | | |  |
|  |  |  |  | 707.26 | 26 |  | | | | | |
|  |  | Daughter ion 519.19 |  | 526.30 | 7 |  | | | | | |
|  |  |  |  | 528.25 | 9 |  | | | | | |
|  |  |  |  | 529.10 | 10 |  | | | | | |
|  |  |  |  | 530.17 | 11 |  | | | | | |
|  |  |  |  | 532.25 | 13 |  | | | | | |
|  |  |  |  | 533.22 | 14 |  | | | | | |
|  |  |  |  | 535.26 | 16 |  | | | | | |
|  |  |  |  | 536.26 | 17 |  | | | | | |
|  |  |  |  | 537.25 | 18 |  | | | | | |
|  |  |  |  | 538.24 | 19 |  | | | | | |
|  |  |  |  | 539.25 | 20 |  | | | | | |
|  |  |  |  | 540.28 | 21 |  | | | | | |
| 7.597 | PMG  C_26_H_32_O_11_ | Major ion  519.20 |  | 520.29 | 1 |  | | | | | |
|  |  |  |  | 521.26 | 2 |  | | | | | |
|  |  |  |  | 522.27 | 3 |  | | | | | |
|  |  |  |  | 523.29 | 4 |  | | | | | |
|  |  |  |  | 524.29 | 5 |  | | | | | |
|  |  |  |  | 526.29 | 7 |  | | | | | |
|  |  |  |  | 527.28 | 8 |  | | | | | |
|  |  |  |  | 528.28 | 9 |  | | | | | |
|  |  |  |  | 529.32 | 10 |  | | | | | |
|  |  |  |  | 530.31 | 11 |  | | | | | |
|  |  |  |  | 531.29 | 12 |  | | | | | |
|  |  |  |  | 533.21 | 14 |  | | | | | |
|  |  |  |  | 535.27 | 16 |  | | | | | |
|  |  |  |  | 537.33 | 18 |  | | | | | |
|  |  |  |  | 538.29 | 19 |  | | | | | |
|  |  |  |  | 541.31 | 22 |  | | | | | |
|  |  |  |  | 543.32 | 24 |  | | | | | |
|  |  | Daughter ion 357.13 |  | 357.28 | 0 | - | | | | | |
|  |  |  |  | 359.23 | 2 |  | | | | | |
|  |  |  |  | 361.16 | 4 |  | | | | | |
|  |  |  |  | 362.30 | 5 |  | | | | | |
|  |  |  |  | 364.30 | 7 |  | | | | | |
|  |  |  |  | 365.28 | 8 |  | | | | | |
|  |  |  |  | 366.28 | 9 |  | | | | | |
|  |  |  |  | 367.31 | 10 |  | | | | | |
|  |  |  |  | 368.29 | 11 |  | | | | | |
|  |  |  |  | 370.25 | 13 |  | | | | | |
|  |  |  |  | 374.32 | 17 |  | | | | | |
|  |  |  |  | 376.30 | 19 |  | | | | | |
| 9.736 | Pin  C_20_H_22_O_6_ | Major ion  381.1 |  | 390.2 | 9 |  | | | | | |
|  |  |  |  | 391.2 | 10 |  | | | | | |
|  |  |  |  | 392.2 | 11 |  | | | | | |
|  |  |  |  | 393.2 | 12 |  | | | | | |
|  |  |  |  | 394.2 | 13 |  | | | | | |
|  |  |  |  | 396.2 | 15 |  | | | | | |
|  |  |  |  | 397.2 | 16 |  | | | | | |

The abbreviations in the table mean phenylalanine (Phe), cinnamic acid (Ca), *p*-Coumaric acid (*p*-Co ), pinoresinol (Pin), pinoresinol monoglucoside (PMG) and pinoresinol diglucoside (PDG).

^13^C stable isotopes were signed by coloured dots. Among the coloured dots, red dots mean that the ^13^C stable isotopes were tracing to the Phosphoenolpyruvate (PEP) converted from ^13^C isotopes labeled glucose, blue dots mean that the ^13^C isotopes were tracing to Enthrose 4-phosphate converted from ^13^C isotop labeled glucose, green dots means that the ^13^C stable isotopes were converted from another ^13^C isotopes labeled glucose through the intermediate substances of PEP as shown in Figure 10 A,B.
